# Hemophagocytic Lymphohistiocytosis Secondary to Disseminated Histoplasmosis

**DOI:** 10.7759/cureus.84241

**Published:** 2025-05-16

**Authors:** Mackenzie Caruthers, Shaili Kothari, Conor Kelly, Kim M Jordan, Branden Luna

**Affiliations:** 1 Internal Medicine, OhioHealth Riverside Methodist Hospital, Columbus, USA; 2 Critical Care Medicine, OhioHealth Riverside Methodist Hospital, Columbus, USA

**Keywords:** dexamethasone, etoposide, histoplasmosis, hyperinflammatory, lymphohistiocytosis

## Abstract

Hemophagocytic lymphohistiocytosis (HLH) is a rare hyperinflammatory condition resulting in erroneous activation of the immune system. Treatment is directed at the underlying pathology that prompts activation of the immune system and usually includes immunosuppressant therapy, including steroids, etoposide, or rituximab. However, the best treatment for patients with significant infection remains unclear. Few cases of HLH are associated with disseminated histoplasmosis, and there are no clear treatment guidelines in these cases. Due to the significant morbidity and mortality associated with HLH, further investigation is needed to identify the best treatment regimens.

## Introduction

Hemophagocytic lymphohistiocytosis (HLH) is a rare hyperinflammatory condition resulting in erroneous activation of the immune system [1,2]. Diagnosis of HLH is based on a combination of serum and bone marrow laboratory findings. Treatment is directed at the underlying pathology that prompts activation of the immune system and usually includes immunosuppressant therapy, including steroids, etoposide, or rituximab [3]. However, the best treatment for patients with significant infection remains unclear, as there are few documented cases of survival associated with the use of immunosuppressive therapies in the setting of acute infection. Here, we describe a case of HLH secondary to disseminated histoplasmosis that was treated with dexamethasone, with plans to initiate etoposide. Ultimately, the patient’s family elected to withdraw support prior to etoposide initiation, and the patient expired. Few cases of HLH are associated with disseminated histoplasmosis, and there are no clear treatment guidelines in these cases [3]. Due to the significant morbidity and mortality associated with HLH, further investigation is needed to identify the best treatment regimens. 

## Case presentation

A 56-year-old female presented to an outside hospital with two weeks of skin discoloration, subjective fevers, and generalized fatigue. Her medical history included psoriatic arthritis treated with adalimumab and methotrexate and recent streptococcus pharyngitis treated with amoxicillin. Computed tomography (CT) imaging was notable for multifocal pneumonia and hepatosplenomegaly. Laboratory values and reference ranges are demonstrated in Table 1.

**Table 1:** Laboratory values on admission with corresponding laboratory-specific reference ranges. H, high; L, low

Laboratory Test	Patient Value	Reference Range
Serum Creatinine	2.94 mg/dL (H)	0.4-1.10 mg/dL
BUN	63 mg/dL (H)	8-25 mg/dL
AST	556 U/L (H)	0-35 U/L
ALT	135 U/L (H)	0-35 U/L
Hemoglobin	8.0 g/dL (L)	12.0-16.0 g/dL
Platelets	47 K/mcL (L)	150-400 K/mcL
Triglycerides	277 mg/dL (H)	30-150 mg/dL
Fibrinogen	38 mg/dL (L)	224-483 mg/dL
Ferritin	>100,000 ng/mL (H)	13-150 ng/mL
Schistocytes	1-2 schistocytes per 1000x immersion field (H)	None detected
INR	2.4 (H)	0.8-1.1
D-Dimer	5.00 mcg/mL FEU (H)	0.27-0.49 mcg/dL FEU

Physical examination revealed an acutely ill woman with jaundice and sclera icterus present bilaterally. Lungs were clear on auscultation with no abnormal heart sounds. Vasopressors were initiated for hemodynamic instability, and broad-spectrum antibiotics were started for septic shock. Given the developing multisystem organ failure and concern for HLH, the patient was transferred to a tertiary care center.

Hematology consultants recommended a bone marrow biopsy to evaluate pancytopenia and the possible initiation of etoposide, rituximab, and dexamethasone therapy pending bone marrow cytology results. An extensive infectious and pancytopenia work-up revealed a histoplasmosis yeast antibody titer of 1:320.

She received liposomal amphotericin B for disseminated histoplasmosis and continued high-dose dexamethasone for persistent hemolysis. As the final pathology from the bone marrow was pending, no immunosuppressive therapy, such as etoposide or rituximab, was initiated. Multiple rounds of balanced transfusions were required for severe pancytopenia and hemodynamic changes, and she eventually required mechanical ventilation. She developed DIC, likely due to septic shock, with decreased fibrinogen, thrombocytopenia, and a prolonged international normalized ratio (INR). On hospital day 3, continuous renal replacement therapy was required for anuria and volume overload following multiple transfusions. Despite antifungal therapy and aggressive support, hemodynamic instability persisted with increasing vasopressor requirements. Arterial blood gas (ABG) analysis showed persistent metabolic acidosis and rising lactic acid, with concern for tissue hypoperfusion despite maximum medical therapies.

**Figure 1:**
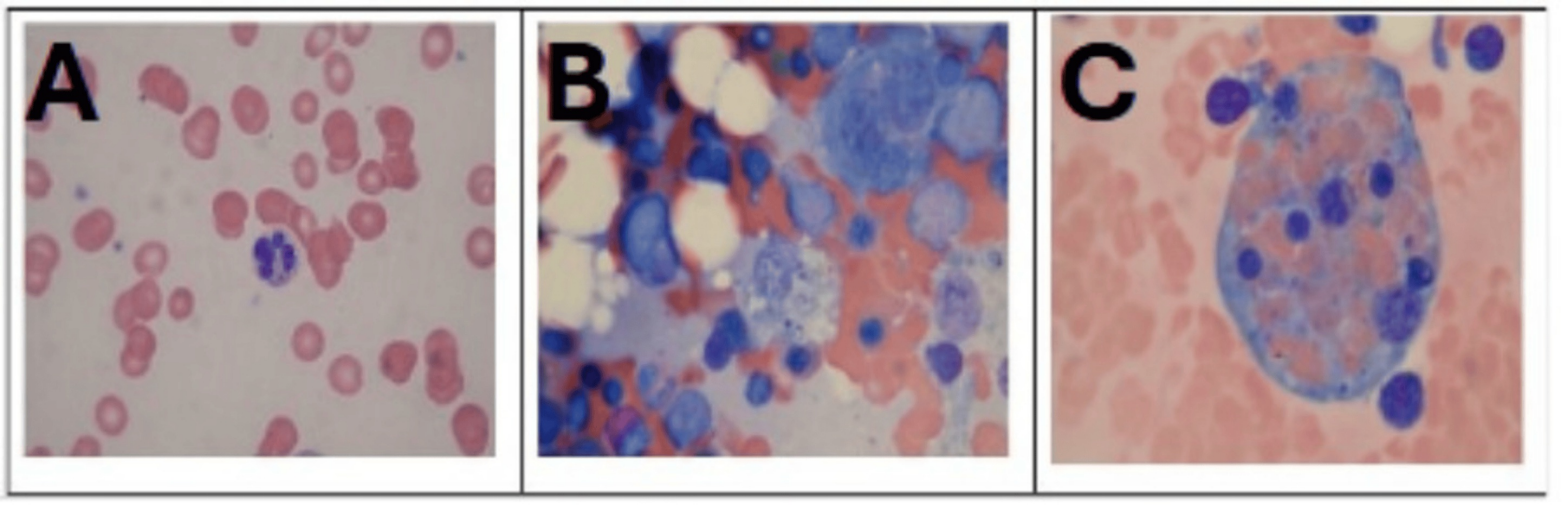
Pathology slides obtained from peripheral smear and bone marrow aspirate. A. Peripheral smear showing a neutrophil containing a yeast form at the 6 o’clock position. B. Bone marrow aspirate smear demonstrating numerous yeast forms within a macrophage just below the center. C. Bone marrow aspirate smear showing hemophagocytosis, with a macrophage containing internalized red blood cells (RBCs) and non-red blood cells (nRBCs).

The final pathology obtained from a peripheral smear showed rare circulating yeast (Figure 1a). On day 4, a bone marrow biopsy confirmed intracellular yeast cells (Figure 1b), consistent with the diagnosis of disseminated histoplasmosis, and macrophages containing both red blood cells and non-red blood cells, consistent with the diagnosis of HLH (Figure 1c). Flow cytometry of the bone marrow aspirate showed 7.8% lymphocytes, of which 80.6% were T-cells and 7.4% were probable NK-cells. Blast cells accounted for 0.7% of events.

As the patient progressed toward multisystem organ failure, the case was re-discussed with the bone marrow transplant team, who recommended initiation of etoposide. However, prior to starting etoposide therapy, the family requested a discussion regarding the prognosis, which led to the initiation of comfort care measures. On hospital day 4, the patient was compassionately extubated and pronounced deceased. A post-mortem soluble IL-2 receptor (CD25) level returned markedly elevated at 53,607 pg/mL (reference range: 175.3-858.2 pg/mL), and an HLH gene panel analyzing 23 HLH-associated genes was negative. Consent for case presentation was obtained from the patient’s husband.

## Discussion

HLH is an aberrant immune response characterized by the proliferation of histiocytes and lymphocytes that inappropriately consume red blood cells. The disease can occur as a primary syndrome in childhood due to underlying genetic mutations or as a secondary condition in the context of malignancy (M-HLH), rheumatologic disorders (R-HLH), immune compromise (IC-HLH), or iatrogenic causes (Rx-HLH) [1]. This hyperimmune state leads to widespread cellular destruction and the accumulation of cell debris in the kidneys, spleen, and liver, resulting in multi-organ failure. Infections are commonly reported triggers of HLH, accounting for 24.3% of cases [2]. Viral pathogens, such as EBV, CMV, HIV, and HSV, are the most frequently implicated, associated with 21.3% of reported cases. Although much rarer, fungal infections, including those caused by Histoplasma spp., are identified as the trigger in approximately 1.8% of HLH cases [2].

The incidence of HLH in adults is not well established, but it has been suggested to be approaching 14 per 100,000 people in the United States. In the largest epidemiological study to date, a retrospective analysis reported that the incidence of HLH increased annually from 2006 to 2019 [3]. Due to limited awareness and evolving clinical diagnostic criteria, the true prevalence of HLH remains difficult to determine. Diagnostic criteria were first established by the Histiocyte Society in 1994, with an update in 2004 and a proposed revision in 2009. The original 1994 guidelines and subsequent updates to the diagnostic criteria are summarized in Table 2 [3].

**Table 2:** Diagnostic criteria from the 1994 HLH protocol published by the Histiocyte Society, with revisions in 2004 and 2009. HLH, hemophagocytic lymphohistiocytosis; XLP, X-linked lymphoproliferative syndrome

1994 HLH Protocol	2004 HLH Protocol Additions	2009 HLH Protocol Additions
Cytopenia	Elevated ferritin	Molecular diagnosis consistent with HLH or XLP.
Fever	Low/absent NK cell activity	At least three of the following four clinical features: fever, splenomegaly, cytopenia affecting at least two cell lines, and hepatitis.
Splenomegaly	Elevated CD25 (IL-2 receptor)	And at least one of the following four findings: hemophagocytosis, elevated ferritin, elevated soluble IL-2 receptor alpha (sIL-2Rα), or absent/markedly decreased NK cell function.
Hypofibrinogenemia or hypertriglyceridemia		Other findings supportive of an HLH diagnosis: hypertriglyceridemia, hypofibrinogenemia, and hyponatremia.
Hemophagocytosis via bone marrow biopsy		

In 2019, the North American Consortium for Histiocytosis recognized that certain conditions can present with a similar spectrum of symptoms, though they would require radically different approaches to treatment. This distinction is made between hyperinflammatory conditions with expected or physiologic immune activation and those with abnormal or destructive immune pathology. The Consortium noted that not all individuals who meet the HLH diagnostic criteria would benefit from immunosuppressive therapy. As a result, it was proposed to use the term "HLH syndrome" for individuals who meet the established criteria but whose immune activation is consistent with the expected course of an underlying condition. The term "HLH disease" should be reserved for the subset of individuals who meet the diagnostic criteria and where immune-related tissue damage remains the primary pathology. This distinction was proposed to clarify therapeutic priorities in patients diagnosed with HLH based on established criteria. In HLH syndrome, the therapeutic priority would be to target the underlying pro-inflammatory condition, while in HLH disease, the focus would be on targeting the immune system with immunosuppressants [1].

Although this distinction is clear, the North American Consortium for Histiocytosis agrees that it remains an area of investigation whether patients who present with sepsis would benefit from immunosuppressive medications in addition to antimicrobials. The decision on how to treat patients with suspected HLH in the setting of disseminated histoplasmosis is further complicated by the fact that disseminated Histoplasma infection is known to cause many hallmark features of HLH (e.g., fever, hepatosplenomegaly, cytopenia), seemingly independent of the disease process itself [4-6]. In the case described above, the patient was treated for HLH syndrome with antimicrobial therapy targeting disseminated histoplasmosis. The decision not to initiate immunomodulating therapy was based on the lack of clear treatment guidelines, as discussed earlier. Antimicrobial medications were used to target the underlying infection, and transfusions addressed the underlying pancytopenia affecting hemodynamic stability.

Following the initiation of antimicrobial therapy, it is proposed that immune-modulating therapy be considered for patients who remain critically ill [7]. The initial treatment protocol published in HLH-94 focused on etoposide-based therapies for familial HLH [8,9]. A 2019 review article compiled 60 cases of histoplasmosis associated with HLH and compared treatment modalities and their outcomes [5]. Since no specific treatment guidelines currently exist, patients in the review were typically started on combination therapy that included etoposide, cyclosporine, high-dose dexamethasone, IVIG, and anakinra, in addition to antifungal therapy. However, only 23 of the 60 patients in the study received HLH-specific medications. Of those who were started on immunosuppressive therapy, 11 survived. Among the survivors, no single immunosuppressive regimen was found to be superior to others. Given the small sample size and documented poor outcomes, the optimal treatment approach for suspected HLH syndrome in the setting of acute infection remains unclear, particularly whether chemotherapeutic agents should be added to therapy targeting the underlying infection.

## Conclusions

Secondary HLH is a dysregulated immune response to an underlying trigger. The treatment of HLH involves addressing the underlying cause of the immune response, along with the use of immunomodulatory therapies. Potential triggers that can initiate this dysfunctional immune pathway include bacterial, viral, and, rarely, fungal infections. Histoplasmosis associated with HLH is extremely rare, and there is limited literature to support any one treatment regimen as superior. Initiation of antifungal medication has been shown to be beneficial; however, the addition of immune-modulating therapies has not been consistently studied as a first-line treatment for HLH. Since this disease requires rapid recognition and prompt initiation of treatment, a process measure to focus on would be the turnaround time for cytology finalization and laboratory results that could impact treatment. Therefore, further investigation is needed to elucidate the efficacy and safety of immunosuppressive agents in patients with HLH secondary to underlying fungal infections.
